# Genomic Sequence Is Highly Predictive of Local Nucleosome Depletion

**DOI:** 10.1371/journal.pcbi.0040013

**Published:** 2008-01-25

**Authors:** Guo-Cheng Yuan, Jun S Liu

**Affiliations:** 1 Department of Biostatistics, Harvard School of Public Health, Boston, Massachusetts, United States of America; 2 Department of Biostatistics and Computational Biology, Dana-Farber Cancer Institute, Boston, Massachusetts, United States of America; 3 Department of Statistics, Harvard University, Cambridge, Massachusetts, United States of America; Broad Institute of Harvard and MIT, United States of America

## Abstract

The regulation of DNA accessibility through nucleosome positioning is important for transcription control. Computational models have been developed to predict genome-wide nucleosome positions from DNA sequences, but these models consider only nucleosome sequences, which may have limited their power. We developed a statistical multi-resolution approach to identify a sequence signature, called the N-score, that distinguishes nucleosome binding DNA from non-nucleosome DNA. This new approach has significantly improved the prediction accuracy. The sequence information is highly predictive for local nucleosome enrichment or depletion, whereas predictions of the exact positions are only modestly more accurate than a null model, suggesting the importance of other regulatory factors in fine-tuning the nucleosome positions. The N-score in promoter regions is negatively correlated with gene expression levels. Regulatory elements are enriched in low N-score regions. While our model is derived from yeast data, the N-score pattern computed from this model agrees well with recent high-resolution protein-binding data in human.

## Author Summary

A eukaryotic genome is packaged into chromatin. The chromatin not only makes it possible to fit the relatively long genome into a tiny nucleus, but also plays an important regulatory role. The nucleosome is the fundamental repeating unit of chromatin. High-resolution tiling array experiments have shown that many nucleosomes are well-positioned in vivo, consistent with an important regulatory role. However, the mechanisms that determine nucleosome positioning are still poorly understood. We have developed a novel computational method for predicting nucleosome positions using only the genomic sequence information. The method detects periodic sequence signatures that discriminate nucleosome sequences from linker sequences. We show that this approach has significantly improved predictive power compared to previous studies. Interestingly, the most predictable regions tend to be located where stringent regulations are needed, i.e., the neighborhood of a transcription start site. This model predicts that nucleosome occupancy is not strongly controlled by short DNA sequence motifs but rather progressively controlled by regular organization of short elements into periodic patterns. We also provide evidence that sequence specificity for nucleosome binding is conserved from yeast to human.

## Introduction

In eukaryotic cells, nucleosomes play important roles in diverse biological processes, including transcription control, DNA replication and repair [1]. The positions of nucleosomes must be well-coordinated in order to ensure proper control of these activities. The coordination of nucleosome positions is a complex process involving interactions among DNA, transcription factors, histone modification enzymes, and chromatin remodeling complexes. How nucleosome positions are exactly determined by these various factors is still poorly understood. Recent genome-wide experiments have identified high resolution nucleosome positions in yeast [2–8], Caenorhabditis elegans [9], *Drosophila* [10], and human [11–13]. These data offer unprecedented opportunities to investigate the regulation of global nucleosome positions.

Of the multitude of factors that regulate nucleosome positions, the role of DNA sequence specificity is of particular interest [14,15]. On one hand, the regular spacing of dinucleotide sequences has the thermodynamic property that favors local DNA bending required for nucleosome packaging [16–19]. Also, certain short DNA sequences have been found to be associated with nucleosome positioning [5,20]. On the other hand, deletion of some promoter sequences seems to have little impact on nucleosome positioning [21]. Random DNA sequences seem to be as competitive for nucleosome binding as genomic sequences in vitro [22]. Another important complication is that nucleosome positions are also altered by many regulatory proteins whose activities do not appear to be sequence specific [23].

Whereas it is difficult to use genetic methods to directly test sequence requirement for nucleosome positioning in a large scale, computational methods that extract sequence features associated with nucleosome binding may offer valuable insight. In addition, as high-resolution mapping of genome-wide nucleosome positions is still experimentally costly, an accurate in silico prediction of nucleosome positions also helps downstream functional analyses, such as improving prediction accuracies of transcription factor binding sites [24]. Three computational methods have been developed recently to predict genome-wide nucleosome positions in Sacchromyces cerevisiae from the genomic sequence [7,25,26]. The first two methods characterize the pattern of nucleosome sequences by counting dinucleotide frequencies, and scan through genomic sequences for matches with this pattern, whereas the third study searches sequence patterns by using k-mer enumeration (k from 1 to 6). Using the tiling array data in [6] as validation, all three groups have found that their methods have a significantly higher predictive power than random guessing, which demonstrates that the nucleosome positioning is not random but partially encoded in the underlying genomic sequences.

However, the fact that the prediction is only modestly higher than random guesses suggests that there may be additional long-range (>100 bp) or sequence independent signals that are important for nucleosome positioning undiscovered by current models. In this paper, we report a novel approach for nucleosome positioning prediction focusing on long-range sequence information. Our method first makes use of the wavelet transformation to extract periodicity features and then uses a statistical model to select features associated with nucleosome positioning. We show that, although still far from being perfect, our model has a significantly improved performance relative to the Segal [7] and Ioshikhes [25] studies in discriminating nucleosome and linker sequences as well as for genome-wide nucleosome positioning predictions. The Peckham study [26] was published while this paper was being reviewed. We show that its performance is similar to ours.

Using the computational model we developed, we were able to predict in vivo nucleosome-enriched or -depleted regions, the negative correlation between promoter nucleosome occupancy and global transcription rates, and the depletion of nucleosomes at regulatory elements. We also predicted that mutation of short DNA sequences only leads to gradual changes of nucleosome occupancy. Finally, we applied the model derived from the yeast data to analyze human genomic sequences and observed a surprisingly good agreement with the experimental data.

## Results

### A Robust Model for Discriminating Nucleosomal and Linker Sequences

Our approach differs from the Segal [7] and Ioshikhes studies [25] in two main aspects. First, instead of characterizing each aligned nucleotide positions independently using a position-specific scoring matrix, we applied a wavelet analysis to extract spatially periodic signals. Second, instead of extracting information from nucleosomal DNA sequences only, we used a logistic regression model to identify signals that help differentiate nucleosome and linker sequences.

Like our model, the recent Peckham study [26] also used a discriminative approach. On the other hand, there are important differences between Peckham's model and ours. First, while Peckham et al. searched for over-represented short sequences (word length ranges from 1 to 6), we targeted distinct periodic signals. Second, we used different statistical models to capture sequence features. Compared to support vector machines, our logistic regression model is easier to interpret.

We analyzed a dataset containing 199 nucleosome sequences [7] and 296 long linker sequences [6] previously identified through high-resolution experiments. The nucleosome and linker sequences were separately aligned as in [7] (see Materials and Methods for more details). Each sequence was converted to 16 numerical signals corresponding to different position-specific dinucleotide frequencies. The dinucleotide frequencies over three neighboring base pair positions were averaged. Each of the 16 numerical signals was then wavelet-transformed.

Fourier and wavelet analyses have been widely used in science and engineering for extracting periodic signals [27]. A major limitation of the traditional Fourier analysis is that it can only detect global periodicities, i.e., those that persist throughout the entire sequence. On the other hand, wavelet analysis is desirable for detecting potentially important periodicities over multiple scales, which is more suitable for our task. In wavelet analysis, a signal is decomposed into orthogonal components, called wavelets, which correspond to the projection of the signal to different frequency bands. In our case the orthogonal components are the Haar bases (see Materials and Methods for details).

The wavelet energies, defined as the total variation at each length scale, characterize periodic patterns embedded in a dinucleotide frequency signal. We reasoned that if a particular frequency is significantly associated with nucleosome positioning, it can be detected by comparing the corresponding wavelet energies for nucleosome vs linker sequences. With this motivation, we model the probability of a sequence being nucleosomal as the logit of a linear combination of wavelet energies (covariates). There are 128 covariates in total. We further used a stepwise logistic regression method to reduce the number of covariates from 128 to 17, which helped alleviate overfitting. We defined the N-score as the logit predicted from this model. More details can be found in Materials and Methods.

### Validation of the N-Score Model

**Figure 1:**
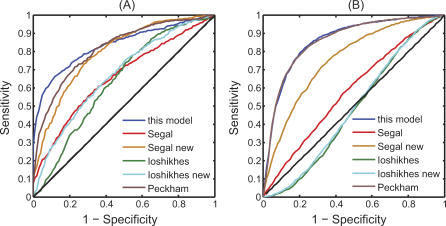
Comparison of the Performance of the Nucleosome Scores from Different Models “This model” refers to the N-score in this paper; “Segal” refers to the apparent free energy score in Segal et al. [7]; and “Segal new” refers to a modified version of Segal's model. The modified apparent free energy score is the log-ratio of the likelihoods of the nucleosome model and the linker model; “Ioshikhes” refers to the NPS score in Ioshikhes et al. [25]; “Ioshikhes new” refers to the same as “Ioshikhes,” except that the NPS pattern was recalculated from the training nucleosome sequences; “Peckham” refers to the support vector machine generated discriminant score using the method in Peckham et al. [26] (A) Cross-validation of model performance in discriminating nucleosome from linker sequences. The plotted ROC curves represent the average performance over five independent rounds of 2-fold cross-validations. (B) Model performance in discriminating nucleosome-enriched probes from -depleted probes in Pokholok et al [4]. The nucleosome scores for (B) are averaged over 300 bp windows.

By fixing the cutoff value of the N-score, we can classify any nucleosome-sized sequence as either a nucleosome or a linker sequence. We compared the performance of different models by using a 5 × 2-fold cross-validation method recommended by Dietterich [28]. The dataset described in the previous section (199 nucleosome and 296 linker sequences) was randomly partitioned into two subsets of equal sizes, with the same proportion of positives and negatives in each training set. Each subset in turn was selected as the training subset with the other reserved for testing. A receiver operating characteristic (ROC) curve was obtained for the testing subset by varying the cutoff N-score values, and the ROC-score, defined as the area under the ROC curve, was used to measure the overall model performance. This 2-fold cross-validation procedure was repeated five times independently. The average ROC curve of our method is shown in Figure 1A, which has an ROC-score of 0.84.

For comparisons, we cross-validated related nucleosome scores derived by other researchers [7,25,26]. For Segal's model [7], the nucleosome score corresponds to the apparent free energy score, which was calculated as described in [7] using the nucleosome sequences in the training subset only. Its average ROC-score is 0.67. For Ioshikhes's model [25], the nucleosome score corresponds to the NPS score. The original NPS score was derived from an independent data source [18], therefore further training was unnecessary. For a fair comparison, we also applied the method in [18] to the training data and re-estimated the NPS pattern (denoted as “Ioshikhes new”). The average ROC-scores for the two procedures are 0.64 and 0.68, respectively. The prediction accuracy of our method was significantly higher than all the three approaches mentioned above (with *p*-values of 0.046, 0.016, and 0.04, respectively, based on the 2-sample t-statistics assuming equal variance; Figure 1A).

To examine whether the performance improvement was mainly due to discriminative modeling or wavelet analysis, we modified the method of Segal et al. to incorporate linker sequence information. More precisely, we center-aligned the linker sequences in the same way as for nucleosome sequences, and then derived a position-specific scoring matrix (PSSM) for the linker sequences in the training set. The modified free energy score was defined as the log-ratio of the likelihoods of the nucleosome model and the linker model. The average ROC-score of the modified Segal method increased to 0.80 (Figure 1A), which is insignificantly lower than that of our model. The fact that the modified Segal model performed better than the original one suggests that the discriminative approach can improve model performance without introducing new sequence features. We also tested Peckham's support vector machine model [26] and found that its performance was similar to ours (average ROC-score = 0.82; Figure 1A). These two examples suggest that the performance improvement we observed was likely due to the discriminative step instead of the specific model employed. Interestingly, while the features utilized by the SVM model, modified-Segal model, and our model are quite different, the performance of these models is remarkably similar.

### The Genome-Wide Prediction of Nucleosome Binding

For the rest of the paper, the N-score model was trained using the pooled data of the nucleosome and linker sequences, excluding the 154 linker sequences that were not in chromosome III. These linker sequences were excluded from the training set since we would test our model predictions on other chromosomes. The final selected variables and weights are reported in Table S1. Using these parameters, we evaluated the N-score for each contiguous 131 bp DNA segment in the yeast genome and assigned this value to its center position. In such a way, we obtained a genome-wide distribution of the N-score, reflecting the predicted sequence preference of nucleosome binding. Genome-wide nucleosome occupancy has been measured in yeast at an average 266 bp resolution [4]. To test whether the above N-score model was able to predict nucleosome-enriched and -depleted regions, we compared the N-scores corresponding to the nucleosome-enriched probes, defined as the 4,000 (about 10%) probes with the highest log-ratio, with the N-scores corresponding to the nucleosome-depleted probes, defined as the 4,000 probes with the lowest log-ratio. To account for the lower resolution, the N-scores over a 300 bp window centered at each probe position were averaged. Remarkably, while the N-score model was trained using information only from the training nucleosome and linker sequences discussed above, the ROC-score is as high as 0.88 (Figure 1B), implying that nucleosome-enriched and -depleted probes can be well predicted based on sequence information alone and that linker sequences are very useful for the identification of such information. In comparison, the ROC-scores for Segal's and Ioshikhes's original models are 0.61 and 0.51 (the results here and thereafter are based on the original NPS pattern in [18]; Figure 1B), respectively. All three discriminative models perform better than the two non-discriminative models. (ROC-score = 0.88 for Peckham's model; ROC-score = 0.78 for the modified Segal model; Figure 1B). The modified Segal model performed slightly worse than the other two discriminative models (Figure 1A and 1B). We suspect this may be due to its sensitivity to sequence alignment errors.

Since measurement errors are inevitable in any experiment, it is desirable to develop a model whose prediction is robust against experimental inaccuracies. The wavelet model is a multi-resolution approach for analyzing spatial signals. When high-resolution nucleosome positioning information is inaccurate, the coarse resolution information may still contain significantly predictive power. To evaluate the impact of experimental inaccuracies on the model predictions, we re-estimated the N-scores by replacing the training nucleosome sequences by those identified from the tiling array experiment [6] (i.e., with 10-fold lower resolution). We observed that the newly estimated N-scores were highly consistent with those derived earlier from the more accurate data, with a coefficient of correlation R = 0.79.

### N-Score Can Reproduce Known Genomic Features of Nucleosome Positioning

**Figure 2:**
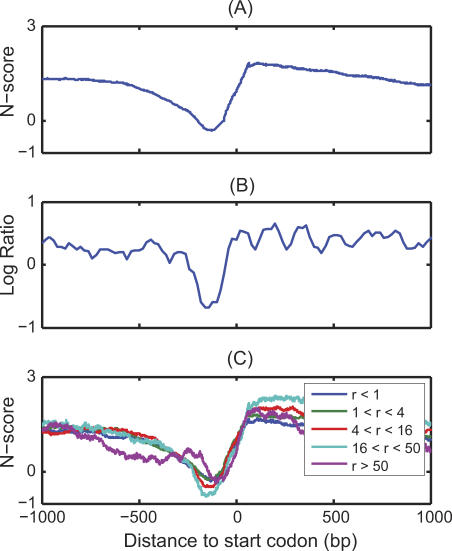
The Average Promoter N-Score Pattern (A) The average N-score pattern over promoters for all verified non–chromosome III genes. Promoters are aligned by the ATG codon. (B) The average log-ratio over non–chromosome III promoters probed by the tiling array [6]. (C) Same as (A), except that promoters are divided into groups according to the gene transcription rate r (in mRNA/h) as in Holstege et al. [29] Different curves correspond to different gene groups.

One of the most striking features of global nucleosome positioning is that most active promoters contain a nucleosome-free region (NFR) near transcription start sites (TSS). This feature has been identified in a number of organisms including yeast [4,6], *Drosophila* [10], and human [11–13]. This overall NFR pattern has also been predicted based on the sequence information by aligning the promoters for all yeast genes at their initial ATG codon and evaluating the average predicted nucleosome occupancy [7,25]. We repeated the analysis but averaged the N-score pattern instead. The results are shown in Figure 2A. Consistent with previous studies, we found good agreement between the average N-score pattern with the experimental data on NFR (Figure 2B). Both the N-score and NFR have a pronounced dip near −200 bp, with a width of about 150 bp. The N-score is noticeably higher in coding than in promoter regions. This is also consistent with the experimentally verified bias of the nucleosome occupancy [3,4]. Compared with the results from [7,25], the N-score curve appears to be smoother and less oscillatory (Figure S1A and S1B).

Genome-wide experimental studies have found that nucleosome occupancy is inversely correlated with gene expression [2–4]. To investigate whether such correlation can be predicted from DNA sequences, we divided genes into five different groups according to their transcription rates from Holstege et al [29]. The average N-scores of genes within each group are shown in Figure 2C. Except for the most active group (r > 50 mRNA/h, purple curve), more highly transcribed genes tend to correspond to deeper N-score valleys in their promoters and more elevated N-score peaks immediately downstream. In addition, we found that the negative Pearson correlation between the promoter averaged N-score and transcription rates is statistically significant (*p* = 3.5 × 10^−4^). This pattern is consistent with previous experimental studies [4]. The pattern is somewhat different for the most active group, which is enriched for ribosomal genes (113 ribosomal genes out of a total of 170 genes in this group). Here, the N-score curve shows a double dip pattern spread over a wider region in the promoters instead of a single deep valley. This double dip pattern also shows up in the curves generated by the other models (Figure S1C and S1D). Whereas the general trends for different models are similar, the N-score curves appear to be smoother. We also repeated the analysis using a more recent and accurate source for genome-wide gene expression [30], and observed a similar result (Figure S1E), confirming that the double-dip feature is not an artifact of any particular choice of gene expression data.

**Figure 3:**
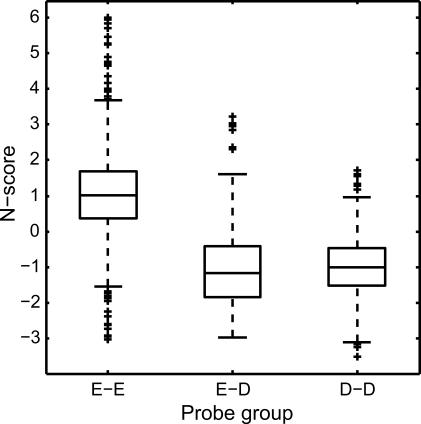
Correlation Between N-Score and H_2_O_2_-Induced Nucleosome Occupancy The box-plot is drawn using the default setting in MATLAB. Coding for the probe groups: E-E, enriched in both YPD and H2O2 growth conditions; E-D, enriched in one but depleted in the other growth condition; D-D, depleted in both growth conditions [4].

Whereas genomic DNA sequence is invariant, the actual nucleosome occupancy is dependent on growth conditions. It is natural to ask whether the genomic sequences in promoter regions associated with conditional nucleosome occupancy are designed to facilitate or exclude nucleosome binding. At normal conditions, the PHO5 promoter is occupied by well-positioned nucleosomes, one of which is centered at −275 bp relative to the ATG codon, occluding Pho4 from binding to its target site at −247 bp [31]. This and three other nucleosomes were depleted upon phosphate starvation, making the Pho4 binding site accessible [31]. Interestingly, this nucleosome is positioned inside an N-score valley from about −400 to about −200 bp, suggesting that the genomic code may be designed to exclude nucleosome binding. To test whether this is a general property for the organization of nucleosomes, we examined the distributions of the N-scores corresponding to the following three groups of probes selected from Pokholok et al. [4] The first group contains probes that are nucleosome-enriched under both YPD and H2O2 growth conditions. The second group contains probes that are nucleosome-enriched under one condition but depleted under the other condition. The third group contains probes that are nucleosome-depleted in both conditions. As shown in Figure 3, the N-scores of the probes in the first group are much higher than those in both the second and third groups, whereas the N-scores corresponding to the second and third groups are indistinguishable from each other. This analysis suggests that the PHO5-like nucleosome positioning regulation may indeed be a general property.

### Effects of Sequence Motifs on Nucleosome Positioning and N-Scores

**Figure 4:**
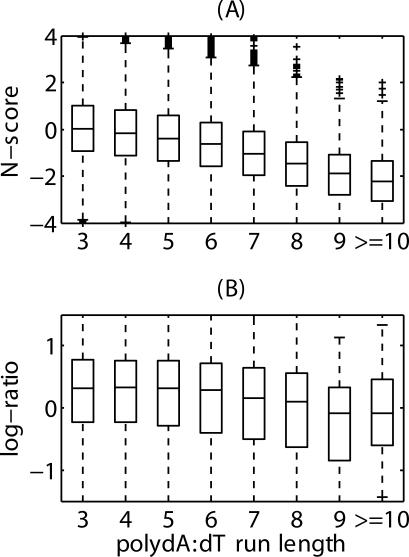
Correlation Between Poly dA:dT Run Length and N-Score, and the BLAST-Entropy Normalized Log-Ratio in Yuan et al. [6] (A) N-score. (B) BLAST-entropy normalized log-ratio in Yuan et al. [6]

A few short sequence features have also been known to be associated with nucleosome positioning. Poly dA:dT tracks destabilizes nucleosomes in vitro and in vivo [32,33]. Recent genomic studies have also associated poly dA:dT with nucleosome-free regions [2,6]. To investigate whether such an association can also be predicted from N-scores, we investigated the relationship between the N-score distribution at poly dA:dT loci (repeat length ≥ 3) in the yeast genome and the dA:dT run length. Figure 4A shows a clear negative correlation between the N-score and the length of its center poly dA:dT track (R = −0.15, *p* < 1.0 × 10^−16^), consistent with experimental results (Figure 4B).

Short elements are unlikely to be dominant factors for nucleosome positions since they are present only at a tiny fraction of sites in comparison to abundant occupancy of nucleosomes and also since histones interact with long stretches of DNA. By thoroughly investigating the regulation of nucleosome positioning at the HIS3-PET56 promoter, Struhl and colleagues examined the deletion of various sequence elements and found that the DNA accessibility gradually increased with the size of the sequence deletion [20]. To test whether this phenomenon can be predicted by N-scores, we conducted the following computational experiment. For *k* = 0, 20, …, 200, we deleted a *k*-bp-long contiguous segment of the promoter sequence (i.e., the intergenic region between HIS3 and PET56) starting from the −1 position of HIS3, and calculated the average N-score of the resulting mutant promoter sequence. Consistent with experimental results, the change of N-score in the promoter region is highly correlated with the deletion length *k* (R = 0.93), increasing from 0.5 to 3.7 as k increases from 0 to 200 bp. Interestingly, Sekinger observed that the nucleosome positions in the HIS3 coding region becomes delocalized as promoter sequences are deleted [20]. This is probably due to thermodynamic competition between neighboring DNA sequences.

To test the generality of this property, we carried out a similar analysis on 100 randomly selected promoter sequences. Each sequence was progressively deleted in steps up to 200 bp. At each step, a 10 bp DNA element was randomly chosen and deleted from the remaining promoter sequence. We compared the average N-score over the truncated promoter sequences with the original ones. Although a deletion may either increase or decrease the average N-score, the change is significantly positively correlated with the length of deletion (R = 0.48, *p* = 2.1 × 10^−119^), suggesting that the progressive effect of short DNA elements on nucleosome occupancy is a global property.

Our systematic approach to analyzing spatially coherent patterns also yields predictions of new sequence features. Table S2 lists all the wavelet components used by our logistic regression model ranked by the significance (*p*-values) of their regression coefficient. Because of our use of a stepwise variable selection procedure, usually only one of two or more highly correlated dinucleotide patterns is included in the final regression model. Therefore, Table S2 is more useful for evaluating the importance of individual factors. Interestingly, we found that the most important predictions appear to be frequencies at the single base pair scale, with TT/AA/TA ranking at the top. AT/AC/GT are also among the most significant predictors, whereas GC seems to only have moderate predicting power. It has been known for a long time that nucleosome binding is thermodynamically favored if the dinucleotides AA/TT exhibit a ∼10.2 bp periodicity. In a wavelet representation, the length scales are discretized and a 10.2 bp periodicity approximately corresponds to 8 bp (level 4) or 16 bp (level 3). As expected, at this frequency band AA/TT are the most significant predictors. Somewhat surprisingly, most of the top ranked sequence features appear to be related to nucleosome exclusion rather than formation, suggesting that the primary role for sequence information may be to establish the boundaries for nucleosome binding.

### Regulatory Elements Are Enriched in Low N-Score Regions

Previous experimental studies have shown that functional transcription factor binding sites are typically nucleosome depleted [2,3], whereas the nucleosome occupancy at unbound motif sites is indistinguishable from the genomic background [6]. To test whether these characteristic differences can be predicted from sequence information alone, we compared the N-score distributions at transcription factor binding sites [34], unbound motif sites [34], and the genomic background. The average N-score is −1.03 at transcription factor binding sites, which is significantly lower than the average over the intergenic regions (−0.77, *p* = 0 from t-test), whereas the average N-score at unbound motif sites is −0.70, similar to genomic background. Therefore, the in vivo nucleosome occupancy differences between bound and unbound motif sites can be partially explained by sequence differences at a longer scale. Similar results have been found in previous studies [7,25] and a comparison is shown in Figure S2. Curiously, for Segal's model, the predicted nucleosome occupancy is higher at both functional and non-functional motifs sites than at the intergenic background.

The TATA box is a universal regulatory element. The promoters of about 20% genes in the yeast genome contain a TATA box [35]. The tiling array data show reduced nucleosome occupancy at the TATA boxes. It is unclear whether this bias is caused by a sequence dependent mechanism, since it was predicted by Segal's model but not by Ioshikhes's model. For our model, we found that average N-score at the TATA boxes is −0.97, whereas the intergenic background is −0.77, and the difference is highly statistically significant (*p* < 10^−100^ from t-test), consistent with the predictions made by Segal et al. The average N-score patterns for the TATA and TATA-less genes are quite similar, though.

### A Hidden Markov Model for Prediction of Genome-Wide Nucleosome Positions

We have shown that the N-score is a useful tool for quantifying the sequence preference of nucleosome binding, but this information needs to be further processed in order the predict nucleosome positions genome-wide. The information summarized by the N-score is similar to that revealed by a chromatin immuno-precipitation (CHIP-chip) experiment, in which the enrichment data need to be processed by computational methods in order to identify protein binding sites [36,37]. In Yuan et al. [6], we have developed a hidden Markov model (HMM) to identify nucleosome positions from tiling array data. Here we used a simplified version of the model. Our HMM structure takes into account the fact that the positions of neighboring nucleosome interfere with each other via steric hindrance. Each hidden state indicates whether or not a position is bound by the nucleosome and follows a Markov chain. The observed variable emitted from the hidden state is the N-score of that nucleotide position. Intuitively, the HMM helps us locate non-overlapping local peaks of the N-scores genome-wide and these peaks represent the predicted nucleosome positions (see Materials and Methods for more details).

**Figure 5:**
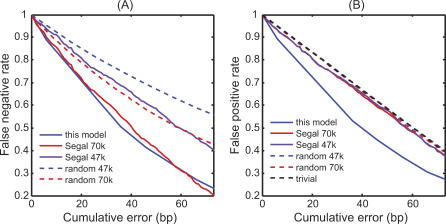
Comparison of the Accuracies of the Predicted Non–Chromosome III Nucleosome Positions Obtained from Segal's [7] and Our Model (A) False negative error rates; (B) false positive error rates. “Random” refers to a random permutation of prediction nucleosomes. “Trivial” means every base pair coordinate is predicted as a nucleosome position. “70k” or “47k” refers to the number of predicted nucleosome positions involved in the comparison. For Segal's model, the top-ranked nucleosomes were selected. Our model predicts a total of 47,000 non–chromosome III nucleosome positions.

In previous studies [7,25,26], the authors quantify their prediction accuracy as the fraction of experimentally verified nucleosomes in a particular genomic region that are correctly predicted by their models, which is equivalent to one minus the false negative error rate. They observed that their model predictions are significantly better than random guessing (i.e., randomly sampling the same number of positions as their model did). We obtained the chromosomal coordinates of the top 70,000 predicted nucleosome positions (of which 1,822 are on chromosome III) from Dr. Segal. The performance of each of the models was evaluated by validating against the non–chromosome III nucleosome positions in [6] because long linkers from chromosome III were used for training the N-score model. With a 35 bp prediction accuracy cutoff (i.e., a correctly predicted site has to be within 35 bp of a true site), the predicted nucleosome positions of Segal's method has a false negative rate of 0.56, compared to 0.66 by random guessing (Figure 5A). Our model has a lower false negative rate of 0.52 despite that it predicted fewer nucleosomes (47,113 in total). For this smaller number of predictions, the false negative rate of random guessing is at 0.75. For a fair comparison, we ranked Segal's predicted nucleosome positions by their predicted probabilities and selected the 47,113 top ranked predictions. The false negative rate of their method was increased to 0.69 due to this reduction of the total number of predictions.

Since the false negative rate is highly dependent on the total number of predicted nucleosome positions, this measure may distort the predictive power of a method. In the extreme case, the trivial prediction that declares every genomic position as a nucleosome position results in a zero false negative rate, but a very high false positive rate. Alternatively, we could measure model performance by its false positive error rate, i.e., the fraction of predicted nucleosome positions that are incorrect, which is insensitive to the number of predicted nucleosomes. At the threshold of 35 bp accuracy, our method have a false positive rate of 0.54, significantly lower than 0.71, the false positive rate of random guessing (Figure 5B). In comparison, the false positive rate corresponding to all predicted nucleosomes from Segal's model is 0.68 compared to 0.71 of the random guessing. For the top ranked predictions from Segal's model, the false positive error is again 0.69. Note that the false positive rate is not sensitive to the number of predictions. For example, the false positive error rate for the trivial prediction is 0.72, which is almost identical to random guessing.

**Figure 6:**
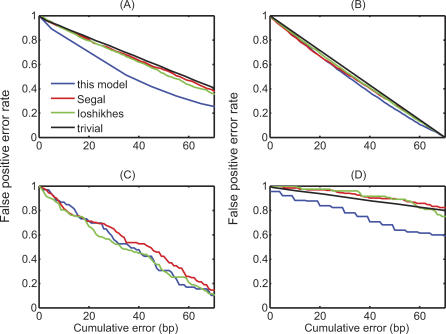
Comparison of the False Positive Error Rates of Predicted Nucleosome Positions Obtained from Different Models (A) Validation with the tiling array data [6]. (B) Validation with the sequencing data [8]. (C) Validation with literature positions as in [7]. Again, a trivial model means every base pair coordinate is predicted as a nucleosome position. (D) Validation of predicted NFR positions with the tiling array data [6]. NFRs are defined as linkers that are longer than 100 bp. Prediction errors are measured by center-to-center distances.

In order to compare with Ioshikhes's model, we downloaded the predicted nucleosome positions from Dr. Pugh's website (http://atlas.bx.psu.edu). Since the model was used to predict nucleosome positions in selected promoter regions only, our validation was further confined to the regions not only probed by the tiling arrays but also by all computational studies. The performances between the Segal and Ioshikhes models for this dataset were similar, with false positive rates at 0.66 and 0.66, respectively, whereas our model had a false positive error rate of 0.51 (Figure 6A).

Peckham et al. predicted nucleosome positions corresponding to 188 contig regions interrogated by Yuan et al.'s tiling array [6]. This was done by applying the HMM in [6] to the nucleosome scores calculated by their support vector machine model. The false positive error rates corresponding to all the predicted positions are shown in Figure S4. At the threshold of 35 bp accuracy, their false positive error rate is 0.57, which is better than the non-discriminative models but slightly worse than ours.

We examined the genome-wide H2A.Z positions obtained recently by Albert et al. [8], who identified ∼40,000 nucleosome positions at a ∼4 bp resolution by pyrosequencing nucleosome DNA. These authors tested the computational models in [7,25] and found that both performed poorly. We applied our method and found that it had negligible improvement over previous methods and still performed poorly (Figure 6B). In addition, we retrained the N-score model using H2A.Z nucleosome sequence information from Albert et al., yet it does not lead to a higher prediction accuracy (Figure S3). Albert et al. found that the average dinucleotide frequency patterns over all their mapped H2A.Z nucleosome sequences are quite different from the usual periodic pattern, whereas only when they selected a small subset of most highly stable H2A.Z nuclesomes (∼8,000 in total) did the periodic pattern re-emerge. It is unclear what other aspects of this dataset may have caused all the methods to fail.

We also validated our prediction results against the literature nucleosome positions included by Segal et al. [7]. For this dataset, our model performed insignificantly poorer (Figure 6C). Considering that this literature set contains only 99 nucleosome positions, and, as noted in [7], there is a significant variation in data quality and growth conditions among different studies. The performance differences might be simply due to random fluctuations. However, it may also be true that each model better predicts some but not all regions, and that a combination of different models may lead to a better overall result.

We next tested on the problem of predicting NFR locations. Here again, we used non–chromosome III data from Yuan et al. [6] for validation. Figure 6D shows that our method more accurately predicted the NFR locations than the other two methods and was significantly better than random guessing. In this exercise, the NFRs were defined as linkers that are 100 bp or longer, and the prediction accuracy was defined as the center-to-center distance between predicted and experimentally identified NFRs. This metric is insensitive to the length variation of either true or predicted NFRs. The high error rate of NFR predictions can be partially attributed to the variability of the width of an NFR. Although the false positive rate for NFR predictions is higher than that for nucleosome position predictions, the relative improvement over random guessing is comparable.

While predictions of genome-wide nucleosome positions are only modestly better than random, it is unclear whether the predictability is uniformly distributed or there exist specific loci that may be associated with exceptionally high predictability. We tested whether the prediction accuracy is dependent upon the distance between predicted nucleosomes and the 5′-end of non–chromosome III genes. We found that the 5′-region is more predictable (false positive error rate at 0.48) than other regions (false positive error rate of 0.52). We repeated the analysis by using the other models and found similar results. For Segal's model, the 5′ region has a false positive error rate at 0.63, compared to 0.71 in other regions; for Ioshikhes's model, the error rates are 0.60 and 0.74, respectively.

### N-Score Analysis for the Human Genome

**Figure 7:**
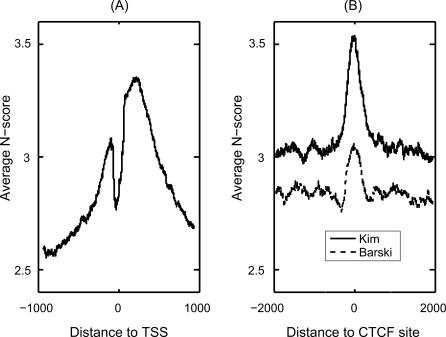
Application of the N-Score Model Derived from the Yeast Data to the Human Genome (A) The average N-score pattern for all human promoters aligned by TSS [39]. (B) The average N-score pattern aligned by CTCF binding sites [13,38].

Since the nucleosome is conserved across all eukaryotes, an intriguing question is whether the sequence preference for nucleosome binding is also conserved. Previous studies have found that the nucleosome sequence patterns in chicken and yeast are similar [7]; however, it is unclear whether the information in non-nucleosome DNA, which is critical for the prediction of local nucleosome depletion as demonstrated above, is also conserved. Recently, high-resolution genome-wide binding sites of several important histone related proteins in human have been experimentally identified [13,38]. To test whether our model predictions agree with these data, we calculated the average N-score at the human promoter regions aligned by known transcription start sites (TSS) obtained from DBTSS [39], where the model parameters for the N-score computation are kept as those estimated from the yeast data (Figure 7A). The ∼100 bp wide dip at TSS and the two peaks located at approximately −100 bp and +200 bp agree well with the locations of the experimentally identified NFRs and adjacent nucleosomes (Figure 2B and 2L in Barski et al. [13]), strongly suggesting the conservation of sequence specificity of nucleosome binding DNA across eukaryotes.

CTCF is a protein that binds to insulators in vertebrates. Recently, CTCF has drawn considerable interest and its genome-wide binding sites in human have been experimentally identified [13,38]. CTCF is also known to be associated with a number of active epigenetic marks, including H3K4me1–3 and H2A.Z [13]. To test whether these distinct epigenetic profiles are related to the sequence-dependent nucleosome occupancy, we computed the average N-score profile for the DNA sequences aligned by the center positions of the reported CTCF binding sites [13,38]. We observed a ∼500-bp-wide N-score peak centered at the CTCF binding sites (Figure 7B), suggesting that CTCF and nucleosome binding share similar sequence specificity. This is consistent with the observation that the H2A.Z also peaks at the CTCF binding sites [13]. Kim et al. [38] have discovered a 20-bp-long sequence motif. Interestingly, while the majority of CTCF binding sites share this motif, there are also a large number of binding sites that do not. Our result suggests that longer DNA sequence information may play a role in the regulation of CTCF binding.

## Discussion

We have developed a novel computational approach for nucleosome positioning prediction and shown that this approach outperforms previous models [7,25] in terms of the ability to recognize known nucleosome and linker DNA sequences as well as to predict genome-wide nucleosome positions. Our method, which uses only sequence information, predicts many properties of in vivo nucleosome positioning, suggesting that sequence information is critical for the overall organization of nucleosomes in living cells.

We have found that our model performs similar to two alternative discriminative models: the support vector machine model recently developed by Peckham et al. [26] and a modified Segal model developed by us. Therefore, the prediction improvement is primarily due to a systematic use of the linker sequence information. The advantage of a discriminative model over a generative model is that it selects features that are specific to either nucleosome or linker sequences, whereas features common to both types of sequences, such as the frequency of the dinucleotide CC, are filtered out. Interestingly, while the other two methods do not explicitly take periodicity into account, the importance of periodicity is reflected in their results (see Figure 1B in [7] and Figure 6 in [26]).

It is interesting to note that predictions of genome-wide nucleosome positions are only modestly better than random guesses, consistent with the conclusion in Peckham et al. [26] Although it is imaginable that further improvement of the computational models may uncover new sequence information that substantially increases the prediction accuracy, another possibility is that exact nucleosome positions may be further fine-tuned through interactions with chromatin modifying complexes and transcription factors, which are environment and stage dependent, and cannot be accurately be predicted from sequence information alone.

While our model was inferred from yeast data, the resulting N-score formula can be used to predict nucleosome occupancy in other species. It is somewhat surprising to us that the yeast-derived N-score patterns of the human genome agree so well with the newly available human nucleosome occupancy data, suggesting that the sequence specificity of nucleosome binding is conserved across eukaryotes.

Typical computational approaches for identifying protein-DNA binding sites assume that the binding only involves short regulatory elements (see Ji and Wong [40] for a review). Whereas these computational methods have successfully predicted transcription factor binding sites from bacteria to mammalian systems, false positive rates of these computational predictions are still very high. Whereas long-regulatory elements have been investigated by combinations of specific DNA binding sites [41–44], the wavelet method proposed here directly detects long regulatory sequences. Our observations here suggest that some overall features of the neighboring DNA sequences of a binding site may be the key to improve protein-DNA binding site predictions.

Our current model ignores several factors that may affect nucleosome positioning predictions. As with previous methods, our model only considers dinucleotide frequencies, whereas longer oligonucleotide sequences may also be important for nucleosome positioning In addition, only the amplitude information of a periodic signal is retained in the N-score calculation, whereas it may also be useful to retain the “phase” information. It will be a worthwhile future work to study how to quantify information in long oligonucleotide sequences and how to properly incorporate more sequence and phase factors for predicting nucleosome positioning.

## Materials and Methods

### Derivation of the N-score.

High resolution nucleosome and linker sequences have been previously identified experimentally. First, 199 high-resolution nucleosomal DNA sequences have been experimentally identified through sequencing by Segal et al. [7]. The length of these sequences varies from 142 to 153 bp. These nucleosome sequences were aligned as in Segal et al. [7], with both forward and reverse strands included. Second, using a tiling microarray-based protocol, we identified 296 NFRs in the promoter sequences [6]. Each NFR contains a stretch of linker DNA whose length is over 100 bp. These linker sequences were aligned in the same way as the nucleosome sequences. For each nucleosome or linker sequence, the central 131 bp were retained for further analysis. Linker sequences shorter than 131 bp were symmetrically extended in both directions. The reverse strand of every sequence is also added to the dataset. For each of the 16 dinucleotides, say *D*, each sequence *S* = (*s*_1_, …, *s*_131_) was first converted to a 130-dimensional numerical vector with its *j*th entry recording whether or not the dinucleotide in (*s_j_*, *s_j_*_+1_) is the same as *D*. Then, every three consecutive positions are averaged to result in a vector of length 128. Thus, each sequence will result in 16 vectors of 128 dimensions.

Let *f_S_*_,*D*_ (*i*/128), *i* = 1,…,128 be the dinucleotide frequency signal for sequence *S* and dinucleotide *D*. Each of these functions is decomposed into a linear combination of wavelet components as

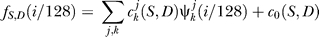
where the wavelet functions 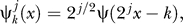 *k* = 0,…, 2*^j^* − 1, *j* = 0,…, 7. Here we used the Haar wavelets; that is,

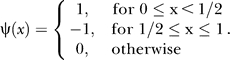



The wavelet coefficients 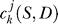 are the projection of the signal 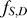 onto 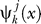 and can be calculated by 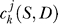 = 
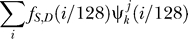
 . Coefficient 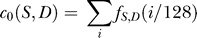 is simply the sum of the dinucleotide frequencies over all positions. The wavelet energy 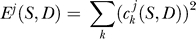 at level *j* measures the total variance of the signal at the 2^7−*j*^ bp length scale. Since there are eight wavelet levels, each dinucleotide gives rise to eight energies for each sequence.

Let *x_l_* (*S*), *l* = 1,…,128 be the collection of wavelet energies at all levels for all dinucleotides. We model the probability *p*(*S*) for *S* being a nucleosome sequence as

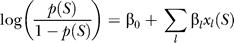



Since the number of predictors is quite large, we used a stepwise procedure to select predictors and estimate the corresponding coefficients using a program in SAS (http://v8doc.sas.com/sashtml) under their default settings.

### Prediction of genome-wide nucleosome positions via a hidden Markov model.

The second step of our prediction model was to take into account the effect of steric hindrance imposed by competing nucleosomes. This was achieved by using an HMM, where the hidden states *H_i_* at the *i*th genomic position are specified by a 2-D vector (B_i_, T_i_), where B_i_ represents whether the genomic position is bound by a nucleosome (B_i_ = N) or not (B_i_ = L), and T_i_ = t, if B_i_ = B_i-1_, B_i-2_, …, B_i-t+1_, but B_i_ ≠ B_i-*t*_. The emission variable O_i_ is the N-score for the 131 bp sequence centered at the genomic position. We assumed that the hidden states have the Markov property: *P*(*H_i_*_+1_ | *H*_1_, *H*_2_, …, , *H*_i_) = *P*(*H_i_*_+1_ | *H_i_*), whereas the successive values of the emission variable are independent from each other. While this model is applicable at a single base pair resolution, this is computationally costly. For numerical simplicity, we divided each chromosome into 10 bp wide bins, and the N-scores within each bin were averaged. The HMM was formulated in terms of the bins, where *i* refers to the bin index. Each nucleosome position corresponds to 15 consecutive bins, whereas a linker may have variable length corresponding to 1 up to 30 bins. The maximum linker length allowed in this model is 300 bp. These constraints can be expressed mathematically as follows.

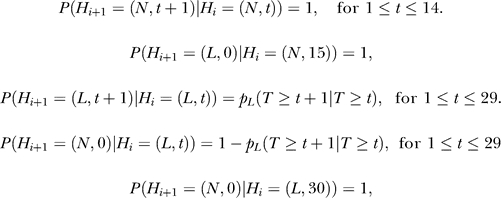
and 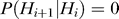 , for other pairs of 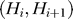 . In the above, 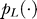 represents the probability distribution of linker length, and it was estimated from experimental data. Finally, the emission distribution P(O_i_ | H_i_) was assumed Gaussian whose mean and variance were dependent only on B_i_ and estimated from the training dataset**.**

### CTCF peaks.

For Figure 7, the center positions of the CTCF sites in Kim et al. [38] were simply the mid-points of the CTCF peaks download from the *Cell* website. The center positions of the CTCF sites in Barski et al. were determined as follows. Counts of sequence tags over 400 bp windows were first obtained from http://dir.nhlbi.nih.gov/papers/lmi/epigenomes/hgtcell.html. Windows containing at least 10 tags were kept. Finally, if two windows were within 5 kb of each other, only the one containing more tags were selected. The purpose of the last step was to select only one window for a CTCF binding site.

## Supporting Information

Figure S1 Comparison of the Average Promoter Nucleosome Score Patterns (A,B) Same as Figure 2A except for using different nucleosome scores.
(C,D) Same as Figure 2C except for using different scores.
(A,C) The free energy score as in Segal et al. [7].
(B,D) The NPS score as in Ioshikhes et al. [25].
(E) Same as Figure 2C except that gene expressions are based on polII occupancy as measured by Dion et al. [30].
z refers to the measured log-ratio.
(1.4 MB EPS)

Figure S2 Comparison of the Average Nucleosome Score Pattern for Transcription Factor Binding Sites (Functional), Non-Functional Motif Sites, and Intergenic Background The nucleosome scores are the N-score (our model), the free energy score (Segal), and the NPS score (Ioshikhes), respectively. For each model, the nucleosome scores are normalized by a multiplicative factor so that the standard deviation is equal to 1. The error bars represent the standard errors of the estimated mean nucleosome score value within each category.
(905 KB EPS)

Figure S3 False Positive Error Rate for the H2A.Z Nucleosome Positions Predicted from the N-Score Model The model was trained by using the H2A.Z nucleosome sequence information obtained from Albert et al. [8]
(527 KB EPS)

Figure S4 False Positive Error Rate of the Nucleosome Positions Predicted by Peckham et al. [26] (605 KB EPS)

Table S1 Selected Wavelet Energy Functions and Corresponding Coefficients from the Stepwise Logistic Regression Model, Using the 199 Nucleosome Squences and 142 Chromosome III Linker Sequences as the Training Dataset A *j*th level coefficient corresponds to variation at the length scale 2^7−*j*^ bp.
(16 KB XLS)

Table S2 Statistical Significance of Wavelet Energy Coefficients in Discriminating Nucleosome and Linker Sequences A *j*th level coefficient corresponds to variation at the length scale 2^7−*j*^ bp. The *p*-value for each wavelet energy coefficient results from a t-test comparing the difference between the nucleosome and linker sequences in the training dataset.
(26 KB XLS)
